# Understanding the Role of Healthy Eating and Fitness Mobile Apps in the Formation of Maladaptive Eating and Exercise Behaviors in Young People

**DOI:** 10.2196/14239

**Published:** 2019-06-18

**Authors:** Mahsa Honary, Beth T Bell, Sarah Clinch, Sarah E Wild, Roisin McNaney

**Affiliations:** 1 School of Computing and Communications Lancaster University Lancaster United Kingdom; 2 School of Psychological and Social Sciences York St John University York United Kingdom; 3 School of Computer Science University of Manchester Manchester United Kingdom; 4 School of Computer Science, Electrical Engineering and Engineering Maths University of Bristol Bristol United Kingdom

**Keywords:** weight loss, mobile apps, eating disorders, body image diet, exercise, mental health

## Abstract

**Background:**

Healthy eating and fitness mobile apps are designed to promote healthier living. However, for young people, body dissatisfaction is commonplace, and these types of apps can become a source of maladaptive eating and exercise behaviors. Furthermore, such apps are designed to promote continuous engagement, potentially fostering compulsive behaviors.

**Objective:**

The aim of this study was to identify potential risks around healthy eating and fitness app use and negative experience and behavior formation among young people and to inform the understanding around how current commercial healthy eating and fitness apps on the market may, or may not, be exasperating such behaviors.

**Methods:**

Our research was conducted in 2 phases. Through a survey (n=106) and 2 workshops (n=8), we gained an understanding of young people’s perceptions of healthy eating and fitness apps and any potential harm that their use might have; we then explored these further through interviews with experts (n=3) in eating disorder and body image. Using insights drawn from this initial phase, we then explored the degree to which leading apps are preventing, or indeed contributing to, the formation of maladaptive eating and exercise behaviors. We conducted a review of the top 100 healthy eating and fitness apps on the Google Play Store to find out whether or not apps on the market have the potential to elicit maladaptive eating and exercise behaviors.

**Results:**

Participants were aged between 18 and 25 years and had current or past experience of using healthy eating and fitness apps. Almost half of our survey participants indicated that they had experienced some form of negative experiences and behaviors through their app use. Our findings indicate a wide range of concerns around the wider impact of healthy eating and fitness apps on individuals at risk of maladaptive eating and exercise behavior, including (1) guilt formation because of the nature of persuasive models, (2) social isolation as a result of personal regimens around diet and fitness goals, (3) fear of receiving negative responses when targets are not achieved, and (4) feelings of being controlled by the app. The app review identified logging functionalities available across the apps that are used to promote the sustained use of the app. However, a significant number of these functionalities were seen to have the potential to cause negative experiences and behaviors.

**Conclusions:**

In this study, we offer a set of responsibility guidelines for future researchers, designers, and developers of digital technologies aiming to support healthy eating and fitness behaviors. Our study highlights the necessity for careful considerations around the design of apps that promote weight loss or body modification through fitness training, especially when they are used by young people who are vulnerable to the development of poor body image and maladaptive eating and exercise behaviors.

## Introduction

### Background and Related Work

Body dissatisfaction, the subjective experience of negative thoughts and feelings toward one’s own body [1], is so prevalent among young people (defined by the United Nations as those aged 15 to 24 years [2]) in modern Western societies that it is regarded as *normative discontent* [3,4]. Body dissatisfaction has been linked with a number of maladaptive eating and exercise behaviors, including restrained eating practices, consuming less fruit and vegetables, low levels of physical activity, excessive exercise, binge-purge cycles, and anabolic steroid use [5,6]. Furthermore, body dissatisfaction is regarded as both an important risk factor for, and is symptomatic of, clinical eating disorders, such as anorexia and bulimia [7,8], the majority of which develop during adolescence and early adulthood [9].

The causes of body dissatisfaction and associated maladaptive eating and exercise behaviors are diverse, with research implicating a combination of biological, psychological, and sociocultural factors [7,10,11]. Sociocultural theories emphasize the role of specific agents, such as parents, peers, and the media, in shaping negative attitudes toward the body [12], with body dissatisfaction arising because of perceived pressure from sociocultural agents to conform to an unrealistic, culturally defined body and beauty ideal. For women, this has been described as thin and toned, yet curvaceous with pert breasts and buttocks, whereas for men it is muscular yet lean with little body fat [13]. The complex and unrealistic nature of this ideal makes it impossible for the majority of young people to achieve, leading to negative feelings around their own bodies [7,12]. In turn, these feelings can motivate an engagement in maladaptive eating and exercise behaviors, aimed at changing the body [7,12].

Perpetuating these social and emotional pressures is the fact that many of these behaviors (eg, clean eating, over-exercising, and cutting out food groups) have become the cultural norm, with magazines and celebrities on social media advocating calorie restriction as an everyday part of how we think about food [14]. Following this longstanding cycle of *diet culture*, parents, who themselves engage in dieting behaviors, can be the ones who convey messages on calorie restriction and *good* versus *bad* foods to children from a young age [15]. Thus, to many young people growing up in this environment, these ways of thinking about food and exercise are seen as the norm and are often engaged with regardless of whether the young person is overweight or not [16]. Ironically, calorie restriction has been demonstrated to lead to weight gain and eating disorders over time in young people [17].

In recent years, the emergence and increasing availability of new digital media and technologies has drastically changed the social landscape. As a consequence, theories of body dissatisfaction and maladaptive eating and exercise behaviors have needed to be adapted. Research in this field has typically focused on how social media influences how young people think, feel, and behave with regard to their body. For example, research has highlighted the role of social media image sharing practices in body dissatisfaction [18]; the further normalization of maladaptive body shaping strategies through user-generated social media content [13]; and the use of social media spaces to create communities centered around maladaptive eating and exercise behaviors [19].

### Healthy Eating and Fitness Apps

The rise of healthy eating and fitness apps is considered to be yet another type of media-influencing body dissatisfaction [20]. The nature of personal mobile engagement itself poses a significant challenge, with vulnerable populations freely accessing content without the extent of their engagement becoming visible to others (eg, a parent who might be unaware of their child’s engagement in calorie-counting practices). However, although there is a wealth of research exploring the dangers of social media use in the development of maladaptive behaviors in young people, comparatively little attention has been paid to healthy eating and fitness mobile apps, despite their ability to become a tool for supporting restrictive eating behaviors (eg, following a 1200-calorie-a-day diet regardless of hunger levels).

The Health and Fitness category accounts for a large proportion of apps in both the Android and Apple app stores (recent statistics place them as the 9th and 8th largest categories of apps, respectively) [21,22]. Although this category includes a variety of health-related domains (including mental health, smoking cessation, and women's health), a significant portion relates to diet, healthy eating, and fitness. In this paper, healthy eating and fitness apps refer to mobile apps that aim to promote healthy eating and fitness (exercise) activities supporting users in monitoring or attending to food and exercise, typically by providing users with additional information about these activities such as calories consumed or burned as well as projections on weight loss goals. The top 10 lists for this category, in both stores, are dominated by apps such as MyFitnessPal [23], Fitbit [24], Strava [25], and Sweatcoin [26], all of which are focused on self-tracking of food or exercise in similar ways.

Healthy eating and fitness apps incorporate a variety of strategies to promote health and fitness behaviors including behavior tracking, goal setting, feedback, rewards, social connectivity, and remote coaching [27]. Adolescent users of Fitbit (a wearable activity monitor with an associated mobile phone app) found that the technology encouraged them to engage in physical activity and increase knowledge of their own health behaviors in the short term [28]. However, there may be a dark side to healthy eating and fitness app use. Many apps are centered on weight loss, encouraging users to set weight-related goals that are achieved through calorie restriction and exercise [28,29]. This emphasis is problematic as the benefits of weight loss to physical health remain a controversial topic within the medical research literature [30,31]. In particular, weight loss is generally only advocated for individuals with high body weight [30], raising concerns about how the cultural preoccupation with weight loss stigmatizes overweight bodies, which may actually demotivate such individuals from engaging in healthy practices in the first place. The growing body of research also shows that body size is not necessarily indicative of good health [31,32]. In addition, calorie restriction has been widely recognized as an ineffective method of weight loss in the long term and an inefficient way of promoting more healthy eating behaviors. Instead, approaches focused on mindfulness and self-awareness, such as intuitive eating, have been advocated more recently [33,34].

The weight loss frameworks that underpin many healthy eating and fitness apps may not be conducive to the formation of positive health and fitness behaviors. In support of this, Eikey et al found that around 7% (1261/18,601) of female app users set weight goals that are under what is considered to be healthy [29], indicative of a desire to achieve unrealistic appearance goals, rather than improved health. The same study found that users felt encouraged by apps to engage in, what the authors considered to be, maladaptive eating and exercise behaviors [29]. For example, some participants described how app feedback following the logging of calories over their set daily target led them to engage in purging behaviors, whereas others described feeling obsessed with thoughts of food and calorie content. Thus, apps focusing on weight loss may inadvertently promote maladaptive eating and exercise strategies.

Research suggests that healthy eating and fitness apps in the personal health domain employ behavior change models because of their potential to support individuals in the process of adapting and maintaining a new healthy behavior [35,36]. The majority focus on habit formation, where habits are defined as automatic responses to contextual cues and are formed as the behavior is repeated in a stable context [37] (eg, using reminders, goal setting, and positive reinforcement). In addition, some apps (eg, Fitbit’s companion app) use competitive techniques such as leaderboards to persuade individuals to be healthier in a playful sense [38,39]. The vast majority of healthy eating and fitness apps implement functionality for the user to self-track, which refers to recording and analyzing data about oneself on a regular basis to monitor a behavior [40]. Self-tracking is largely believed to only be effective if the monitoring continues [41]. Hence, self-tracking can become a system of constant recordkeeping and monitoring, requiring repetitive behaviors [42]. The literature has shown that self-tracking practices, promoted by healthy eating and fitness apps, can have a negative impact on the well-being of young people, by encouraging addictive tracking behaviors and negatively influencing body image [40]. Theoretical work [43] argues that long-term use of digital technologies to monitor health behaviors may objectify the body, encouraging a mind-body dissociation that results in negative affect and the *anaesthetization* of the human experience. Such experiences may prevent the active adoption and ownership of positive health and fitness behaviors [43].

### This Research: Aims and Research Questions

Despite the popularity of self-tracking apps, and the fact that they are largely marketed at younger demographics (with those aged 18 to 29 years being the most regular users [44]), there is a distinct lack of research on young people’s experiences of healthy eating and fitness apps and self-tracking practices [45] and even less so on the potential harm that these self-tracking behaviors might have [46]. Our study attempts to address this gap in the literature by highlighting potential areas for concern. By drawing attention to this issue, we hope to encourage the development of future technologies that are sensitive to the sociocultural pressures surrounding diet and exercise that young people are already contending with.

In this paper, we describe a series of engagements conducted with 106 participants over the course of approximately 12 months. First, we conducted an anonymous Web-based survey (63 male and 32 female) to gather an overview of the types of healthy eating and fitness apps young people are using, their reasons for nonuse, and any maladaptive eating and exercise behaviors that they had noticed in response to app use. We then conducted 2 workshops with 8 young people to gain an insight into their overarching positive and negative experiences of engaging with healthy eating and fitness apps. We conducted a further 3 interviews with experts in the domain of eating disorder and body image to gain a deeper understanding of clinical presentations of maladaptive behaviors and how the use of healthy eating and fitness apps might exasperate such behaviors. Finally, drawing from insights gathered during Phase 1, we conducted a review of the top 100 healthy eating and fitness apps on the Google Play Store (ie, for Android apps) to explore the extent to which they had the potential to contribute to maladaptive eating and exercise behaviors highlighted by participants.

Through this body of research, we aimed to address 4 research questions: (1) What are the experiences of both female and, yet underrepresented, male young people around using healthy eating and fitness apps?; (2) How do experts in body image and eating disorder feel about healthy eating and fitness app use among young people?; (3) To what extent do current healthy eating and fitness apps on the market address, or indeed confound, the issues identified by young people; (4) How might the designers of future tools support young people in their healthy eating and fitness goals to ensure that they are developing these responsibly, without them becoming a potential source of issue, especially for those at risk of maladaptive eating and exercise behaviors.

## Methods

### Phase 1 Methods: Understanding Perceptions and Potential Harm

The research presented in this paper received ethical approval from the research ethics committee of Lancaster University. All participants consented to take part in the study, with survey participants being presented with the study information on a welcome page and workshop and interview participants providing written informed consent.

#### Survey

To begin our study, we first wanted to gain some broad insights into the types of healthy eating and fitness apps that young people currently use, their motivations for using healthy eating and fitness apps, and the reasons they might have for discontinuing use of these types of apps. In addition, we wanted to explore if young people currently reported any maladaptive eating and exercise behaviors associated with their healthy eating and fitness app use, to provide an overarching understanding of the possible issues they might have and to better inform the design of our in-depth workshops.

We created an anonymous Web-based survey, distributed through social media using a snowball sampling method. With acknowledgment that the existing literature around body image and maladaptive eating and exercise behavior centers mainly around cisgender female populations [47], we wanted to, where possible, gather a gender-balanced perspective during recruitment. As such, when we released our call for participation, we noted that we were interested in the views of people identifying as both female and male.

Participating respondents (n=95, 63 male) were asked to follow a link to the survey where they were then asked the following: the gender they identified with, their nationality, whether or not they currently use a healthy eating and fitness app (and if not, why), which apps they used now or in the past, their primary motivation for using these apps, and whether they felt the app(s) caused any negative experiences and behaviors. Responses were collected in both free text and tick box format. After a 4-week period, the survey was closed and responses were collated.

#### Workshops

We next ran 2 workshops to better understand how young people engage with healthy eating and fitness apps and what they perceive to be the dangers of the use of these apps. We wanted to balance the perspectives of both (1) typical users and (2) those advocating for positive body image, who we felt represented current body positive activism taking place in Web-based communities such that that young people may come across in their own social media feed, thus representing a possible positive influence.

##### Workshop Participants

Despite opening our workshops to both genders, the workshop participants were all female. Participants were recruited via an email advertisement. The first workshop was held with 6 female students recruited from York St. John University: 3 studying psychology (W1-P1, W1-P2, and W1-P3) and 3 studying sports sciences (W1-P4, W1-P5, and W1-P6). The workshop was facilitated by 3 researchers. The second was held with 2 *peer educators* from a local youth organization who had undertaken training explicitly around promoting a positive body image in young people (W2-P1 and W2-P2). Peer educators are trained 14- to 25-year-olds who help youth groups explore important topics such as body confidence and self-esteem, using their own experiences to bring the subject to life [48]. The second workshop was facilitated by 2 researchers who were also present in the first workshop.

All participants were aged between 18 and 25 years and had current or past experience of using healthy eating and fitness apps. Participants were provided with a £10 Amazon voucher as a thank you gift for their time. Each workshop lasted approximately 2 hours. Both workshops were audio-recorded and transcribed with the participants' knowledge and consent for qualitative thematic analysis at a later date.

##### Workshop Activities

**Figure 1:**
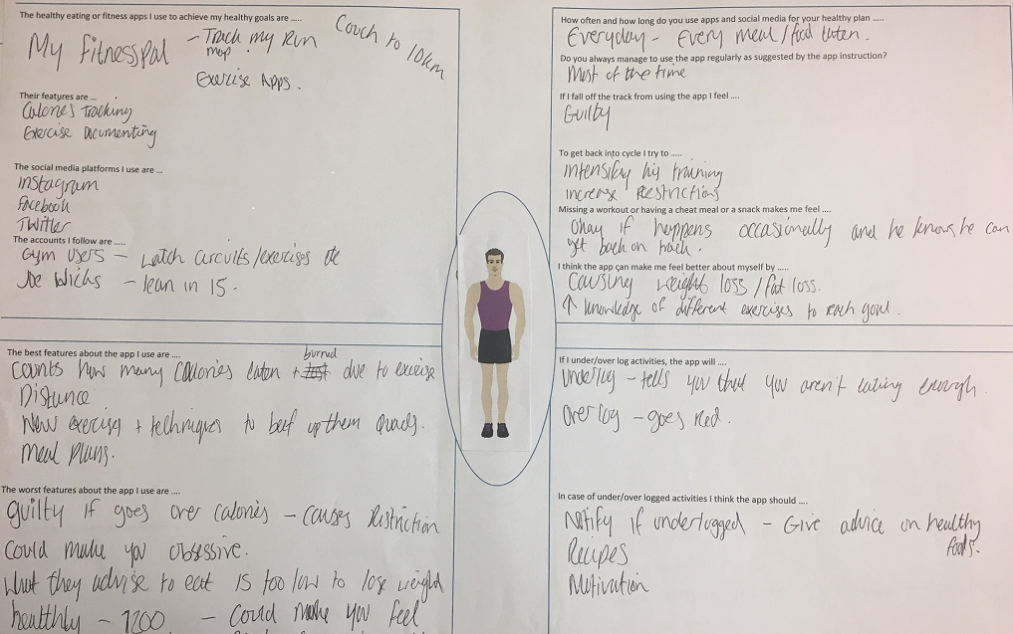
Example of persona activities.

Workshops 1 and 2 both followed the same format. We first facilitated a series of activities designed to encourage participants to talk about their experiences of healthy eating and fitness apps. Owing to the potential sensitive nature of the topic area (ie, maladaptive eating and exercise behavior formation), we asked participants to work with personas to allow for a level of dissociation from their own personal experiences [49,50]. For the first activity, using a provided storyboard sheet, (see Figure 1), participants each created a persona that, to them, represented a person who was engaging with healthy eating and fitness apps. They were prompted to think about their persona’s health experiences, including their health goals, what they considered to be a healthy body, and their strategies for achieving their health goals.

We then randomly split the group into 2 and asked each group to select a persona to work with for the next activity, which aimed to better understand their relationship with apps and their patterns of use (note that workshop 2 participants each worked with their own personas for this activity). Participants were asked to identify the healthy eating and fitness apps they use to achieve their goals and these apps’ features; the duration and frequency of engaging with the apps; whether or not they followed the app’s instruction on usage; how they felt when falling off track when using the app; what they do to get back on track; and how the app might make them feel about themselves. We then wanted to know what happens when they under or over log their activities in the app; the app’s response in this situation; and what they think the apps should do in this scenario.

Following this, the final activity revolved around having a discussion across the whole group about the effect of following a healthy eating and fitness goal and the use of technology to achieve this goal on the individual’s social life. This included the effect on social activities and any impact on friends and families.

#### Expert Interviews

Following our workshops that allowed us to gather young people’s experiences around healthy eating and fitness app usage, we wanted to hear experts’ views on the potential impact of healthy eating and fitness app use on body image and self-esteem within young people. This aimed to allow us to map workshop participants’ experiences to possible negative experiences and behaviors (ie, what *could* happen if unhealthy eating behaviors become more extreme). We carried out 3 in-depth interviews with experts exhibiting a range of eating disorder and body image experiences (from clinical practice, personal experience, and academia): (1) a clinical psychologist with expertise in child and adolescent mental health (E-1); (2) an eating disorders campaigner, activist, and writer with personal experience of eating disorder who advocates for greater recognition of eating disorder service delivery and the male experience (E-2); and (3) an academic researcher in social psychology with expertise in body image, exercise psychology, motivation, and well-being (E-3).

Each interview was conducted in a semistructured manner, with the topic guide centering around the interviewee’s personal or professional experiences with maladaptive eating and exercise behaviors and negative body image; their experience of healthy eating and fitness apps; their views on the impact of healthy eating and fitness apps on young people, including any negative experience and behavior that app use might incite; and their opinions on how future apps might be improved to make them safer to those at risk of maladaptive eating and exercise behaviors. Interviews were all conducted remotely via WebEx and lasted between 30 and 45 min. Each interview was audio-recorded and transcribed for later analysis. Experts were not paid for their time.

### Phase 2 Methods: Reviewing Current Apps

The first stage of our study allowed us to identify any negative experiences and behaviors that young people might have when using healthy eating and fitness apps, and understand how these behaviors might be associated with more extreme maladaptive eating and exercise behaviors. Phase 2 of our research drew upon the increasingly common practice of surveying and evaluating mobile apps in targeted health domains [51-56]. The majority of these previous mobile app reviews have focused on engagement, functionality, aesthetics, and perceived quality, using tools such as the mobile app rating scale [57]. This scale facilitates structured scoring on not only user experience but also on the likely impact of an app on user knowledge, attitudes, intention, and action in the target health behavior. However, as our app review is focusing on the potential issues with such features that, for example, promote engagement or change attitudes toward those deemed as *healthy*, we did not feel this was an appropriate tool for our own review. To the best of our knowledge, the app review conducted in this paper is the first to have the explicit goal of identifying the risks for apps targeting healthy eating and fitness.

We conducted a systematic analysis of the top 100 healthy eating and fitness apps that are currently available on the market for young people to freely download and use. We used content analysis to examine the potential of these apps to elicit the negative experiences and behaviors identified in Phase 1. A breakdown of the review methods is given below.

#### Identifying the Top 100 Apps

We focused our attention on Google Play, the Android app store, because of its market dominance when compared with other operating systems [58]. We first developed a simple tool in Python to allow us to scrape the app store and return the top healthy eating and fitness apps based on a series of filter terms linked to healthy eating and fitness. For eating, there were 20 filter terms in total (eg, calorie, weight, nutrition, and diet), and for fitness, there were 18 terms in total (eg, exercise, fit, flexibility, and training). We focused only on freely available apps as findings have shown that, although young people (aged 18 to 34 years) are more likely to pay for an app than other age groups, the majority still do not pay for any mobile apps at all [59]. Our initial search yielded 540 free healthy eating and fitness apps. We re-ordered the list based on popularity (ie, number of downloads) and then manually reduced the list by disregarding apps that did not match our definition of healthy eating and fitness apps. This included removing apps that were explicitly (1) *wellness*-related (eg, containing the terms yoga, calm, and meditation); (2) route planners for cycling, running, and walking; (3) those that monitor physiological measures (eg, heart rate and blood pressure); and (4) specific to an aspect of health that was not diet or exercise related (eg, menstrual tracking, sleep, drink water reminders, and quit smoking). We then selected the top 100 of the remaining apps for our review. 

#### Review Methods

An app review criteria list was developed based on the themes that emerged from our Phase 1 analysis, reflecting the concerns expressed by experts and users regarding the specific types of logging behaviors facilitated by apps and the way in which apps respond to these behaviors. More specifically, we created a series of reviewing questions that aimed to identify the following:

- General Purpose (Qualitative)
- Data Logging including the types of data entry and behavior logging facilitated by the app (Qualitative)
- Specific Behaviors including the ability to set underweight goals and ability to report low or high calorie consumption (both quantitative: Yes or No) as well as the app responses to these behaviors and other behaviors of interest, including continuous use, not meeting exercise goals, and over and under logging calories (Qualitative)
- Feedback including whether the reviewer perceived the app to rely on mainly positive or negative feedback to motivate users (Qualitative) and whether the app was more focused on what the user had or had not achieved (Qualitative).
- Data Sharing including ability to share data with other users (Quantitative: Yes or No) and across social media (Quantitative: Yes or No) and ability to rank self among other users (Quantitative: Yes or No).

Each app was reviewed by one of the authors who downloaded the app to their personal handset, engaged with the app for a week, and responded to the review questions via a Web-based review form. Apps were downloaded in batches of 10 so that the reviewer was assessing no more than 10 apps at any one time. To assess for interrater reliability of the reviews, a 20% subset of the apps were coded according to the same questions by a research intern, blind to the aims of the study. Responses by the 2 reviewers were then scrutinized by the research team for agreement. For items that generated quantitative data, we followed the guidance of West et al [36] and divided the number of agreements by the number of disagreements. The concordance rate of 88% was comparable with that reported by [36]. For items that generated qualitative responses, these responses were compared for any substantial differences, but none were found. Thus, an agreement between reviewers was found to be acceptable across all review criteria, both qualitative and quantitative.

## Results

### Data Analysis: Phase 1

A content analysis [60] was conducted on the survey data to look for emergent themes relating to each specific question. The qualitative data collected from the 2 workshops and the expert interviews were coded by 2 members of the research team using Braun and Clarke thematic analysis [61] to identify overarching themes from discussions, capturing the core topics and concerns coming from the data. The analysis was inductive and, therefore, data driven. Coders then worked together and discussed any discrepancies before agreeing on a final set of themes. The identified themes were discussed and refined with input from another researcher to ensure that they were representative of the data and could inform the app review process.

### Phase 1 Findings

#### Survey Findings

**Figure 2:**
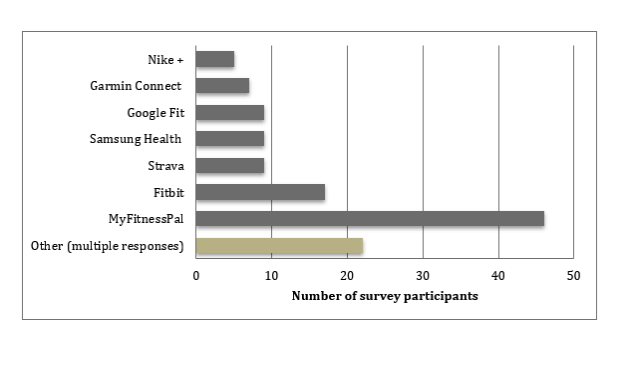
Healthy eating and fitness app use as reported by survey participants. The 7 most popular apps are used by between 5.2% and 48.4% of respondents. A further 23.2% listed other apps (not itemized here because of their limited popularity).

In total, we had 95 respondents who took part in the survey, 63 identifying as male and 32 as female. The majority of respondents were based in the United Kingdom (75), but we also had responses from the rest of Europe (9), United States (6), Canada (3), and China (2). Respondents reported using a range of healthy eating and fitness apps (see Figure 2 for details), but MyFitnessPal, a calorie-tracking diet-focused app, was by far the most widely used (n=46). There were also a wide range of other lesser known apps that individuals (1 to 2 people in each instance) reported using (n=22). Respondents also reported a variety of reasons for engaging with apps (see Table 1 for details). Calorie-tracking (31%), weight loss (20%), and exercise (21%) were the 3 most common reasons. Please note that this was a free text box and, as such, participants could select multiple motivations.

**Table 1:** Reported motivation to engage with healthy eating and fitness apps.

Reason provided	Female, n	Male, n	Total (N=95), n (%)
Track calories	13	18	31 (33)
Track macro nutrient values	4	5	9 (9)
Exercise	10	10	20 (21)
Weight loss	11	8	19 (20)
Track routes or time for specific activities, for example, cycling or running	0	10	10 (10)
Self-improvement (eg, improved body image)	1	10	11 (12)

#### App Nonuse

A total of 32 participants (21 male and 11 female) reported that they no longer used their healthy eating and fitness app (see Table 2 for details—as above, participants could provide multiple responses). Respondents’ primary reasons for discontinuing use of an app were that it was (1) too demanding on their lives, reporting that they were “too busy” or that it took “too much time to track” and (2) that they lacked motivation, discussing *laziness, apathy*, and a *lack of discipline* as contributing factors. Males were more likely to report that they had met health goals, for example, “no longer needed after my weight loss” and “my goal was to get in shape”. Other responses for male respondents focused mainly on challenges with the technology, for example, “Background processes making phone run slower”, where for 2 of the females, it was an eating disorder that caused them to halt their use: “I have an eating disorder and it was toxic”; “Eating disorder recovery”.

#### Reports of Negative Experiences and Behaviors

A total of 41 respondents indicated that they had experienced some form of negative experiences and behaviors through their app use (see Table 3 for details—as the above participants could provide multiple responses). Of those, the most commonly reported behavior across both males and females (n=15) was obsessive calorie counting or logging, with comments such as “I didn’t like MyFitnessPal as this was about counting calories and I became obsessive. It wasn’t manageable”; “I got obsessive over calorie counting”; and “Want to log everything I eat”. There were also reports relating to guilt and food restriction: “constant guilt”; “MyFitnessPal can cause a self-judgment based on bad diet days”; “yes. Starvation”; and around impacts to social life; “Purposefully taking time away from studying and social activities to exercise”; and “stopped socializing (drinking) as much, avoided eating out”. Several respondents discussed general impacts to mood, indicating that they became “miserable”, “anxious”, and “demoralized”. The males in particular also discussed feelings of *disappointment* and *failure* when not achieving their goals.

**Table 2:** Reasons provided for app nonuse.

Reason provided	Female, n	Male, n	Total (N=32), n (%)
App use too demanding on their life	4	5	9 (28)
Lack of motivation	4	5	9 (28)
Health goals were met	0	5	5 (16)
Other	3	3	6 (19)
No response	0	3	3 (9)

**Table 3:** Reports of negative or obsessive app use behaviors from respondents.

Behavior reported	Female, n	Male, n	Total (N=41), n (%)
Obsessive counting or logging	8	7	15 (36)
Guilt and food restriction	3	2	5 (12)
Impact on social life	3	2	5 (12)
Impact on mood	2	5	7 (17)
Cheating the technology	2	0	2 (5)
Obsessive, but the push needed	2	2	4 (10)
Non applicable response	0	3	3 (7)

However, for a handful of respondents, there was a feeling that, although the app led to obsessive exercise behaviors, these were the push that they needed to succeed, forcing them to do exercise when tired: “You have to be a bit obsessive to get good results with a calorie counting app”; “Negative, no. Obsessive, possibly. In the case of exercise, there is aspects of obsession anyway”.

For 2 of the females, there was a discussion of cheating the technology, for example, “I wouldn't enter ‘bad’ foods into the app”, where for 3 of the males the negative feelings they harbored were relating to the technology itself and annoyance that was felt when their effort was not recorded: “Yes - if steps aren’t recorded it feels like they haven't happened”.

#### Workshop and Interview Findings

The following sections describe a synthesis of findings from workshops 1 and 2 and the expert interviews. A total of 4 overarching themes emerged from our thematic analysis: (1) factors relating to motivation and guilt surrounding goal attainment; (2) impact of healthy eating and fitness behaviors on social relationships; (3) app response to logging behaviors, in particular when these might be seen as negative; and (4) a need to reconnect to the body and build self-awareness around healthy versus unhealthy behaviors. We append quotes provided with participant numbers and an indication of whether quotes are from workshop participants (eg, W1-P2) or our experts (eg, E-1).

#### Goal Attainment: Positive Motivation or Guilt Inducing?

Participants discussed a range of healthy eating and fitness apps that they used; however, as can be seen in the data presented, the ones that massively dominated conversation centered around MyFitnessPal and FitBit. There was much discussion around the qualities that these apps had and differences between the active and passive tracking functions they exhibited. All of the participants, in different ways, highlighted how they favored apps that passively track physical activities, compared with ones requiring users to actively log exercise or food intake data. For example, W2-P2 described:

Myfitnesspal is a lot about weight, it’s mainly about logging your food and your calories, whereas fitbit I think is a really positive tool [...] people use them to just increase your daily step count, make yourself just more generally physically active.

Continuing the discussion over active versus more passive tracking, W2-P2 noted how the burden of inputting individual food items, particularly in home-cooked meals, could actually lead to a somewhat counterintuitive shift toward readymade food: “you’d definitely eat more processed things, ‘cos they’re easier to log ‘cos it’s got a barcode” (W2-P2).

Apps that implemented a positive approach to encourage users for their effort were seen to be more appealing. Participants noted that receiving “little badges” (W2-P2) or a simple message such as “nice one, you’ve done that well” (W1-P5) could be *rewarding* or *motivating*. There was a sense that apps should send positive notifications regardless of whether the daily goal was achieved:

[On Fitbit] at the end of the day if you don’t hit your daily step target [...] it just goes back to the next day, but then if you do hit it, it goes green and it’s like “woohoo”. But MyFitnessPal, I think it’s like addictive methods of trying to get you to use it to reach your goals.

W2-P2

However, this concept of achieving set goals was not always seen to be a positive thing. Participants described how fixating around the attainment of a goal could lead to obsessive behaviors: “You don’t want to do badly, so it makes you more obsessive of reaching your goals” (W1-P1) and how not reaching a specific goal could lead to feelings of distress and guilt: “The worst features of the apps are that he [the persona character] feels guilty if he goes over calories and that can cause further restriction and it’s made him obsessive” (W1-P4), which could in turn lead to negative counter-behaviors, such as meal skipping: “you could reach your calories at like 3pm and then you don’t feel like you could eat anything else for that day” (W1-P1).

E-3 described how the prescription of goals—such as hitting 10,000 steps a day—may overshadow any intrinsic motivations for exercising (ie, with focus being placed on reaching a target number rather than factors such as improving fitness or mental clarity):

The monitoring apps can make you think about physical activity in quite a sad way, or quite a compulsive way. If you have a step counter actually you think “I’m going to go for a walk to get my step count up” and your motivation behind doing it is quite external, it’s not about enjoying the walk for example, it’s not about getting some fresh air or just clearing your head for a minute, actually suddenly that walk is all about “well this is going to get me about 3000 steps if I do this 20 minute walk at lunch time” for example.

E-3

E-1 discussed how a focus on these aspects of quantification and goal attainment could become a potential risk for the emergence of maladaptive eating and exercise behavior, if paired with obsessive personality traits or perfectionism:

People who tend to have an obsessive personality and also high levels of perfectionism will sustain their use of fitness apps and count every calorie, count every step, count every episode that they go to the gym and all their exercise and use a mobile platform to collect that data.

He noted how, in clinical care, “we actively discouraged [people with eating disorders] from using those apps and various wrist monitors that count the steps and activity levels” as they can become “triggering” as well as sustaining “obsessional and restrictive and rigid behaviors”. E-2 further highlighted this point by describing his own personal experience of using healthy eating and fitness apps to track his food intake and exercise levels:

I got to this point where I felt like I was really in control and I found the counting of the calories wasreally empowering...especially running and walking [...] you can put it on and it tracks you and it gives you your speed, and I was really fixated on how far I was going and how fast I was going.

E-2

E-2 also highlighted how this eventually led to him losing a sense of control:

I no longer felt in control, it sort of started spiraling and I was losing like five pounds, six pounds.

At this point, he realized that he has established an unhealthy relationship with healthy eating and fitness apps:

It all had to sort of go because I just realized how unhealthy it was and I know that I can’t really use those kind of apps in a healthy way.

#### Impact on Relationships

Participants discussed how heavy dieting and exercise regimes could have a negative impact on maintaining a good social life; many social gatherings focus on food, and often, a lot of time is required to maintain a heavy gym regime. W2-P1 described how, for some people, socializing becomes challenging because of the need to make dietary sacrifices:

If you’re very religiously logging it and then you think oh I can’t go out to that restaurant or I don’t want to go out for drinks.

The concept of religiously monitoring caloric intake in an attempt to lose weight was highlighted by E-1 as a key challenge that could ultimately lead to social isolation and impact of the psycho-social development of a young person:

For a lot of people it becomes an obsessive, all-consuming pursuit and therefore all energy is invested in that and there is no space left to do other things like invest time in social relationships, in social gatherings, education, family, friends and other sorts of developmental aspects of growing up.

It was noted how engagement with apps on a mobile phone is largely, by design, a personal and private experience, which could in fact intensify negative experiences and behaviors, as it is easy to hide from family and friends:

...by the time you’ve noticed it they’re probably already obsessive, because apps are actually very easy to hide that you’re using it

W1-P1

W2-P2 also described how easy it is for young people to engage in secretive behaviors without anyone finding out:

It’s on a phone where you’ve got a password. Sure, people can have visibility of it but they may not even know you have it. So say if it’s a young, like a teenager, their parents may not even know they’re using it, why would they, it’s on a locked phone [...] I think you can quite easily start to like manipulate your calories in a negative way and nobody would know you’re doing it.

#### App Response to Logging Behaviors

Participants discussed the issue of under logging calorie intake and how an app may respond in such cases:

It comes up in red basically giving you a warning that you’re not eating as much, or, if you carry on like this you’ll be losing two pounds in a week.

W1-P2

However, they discussed that providing such weight loss projection could motivate users to eat less to lose weight faster within the estimated time frame. Participants explained how, when the user underlogs, apps currently only respond with a warning notification, instead of taking any actions to prevent this from happening. For example, W2-P2 described how she restricted her calorie intake to 1200 calories per day for 7 months without any guidance from the app to re-evaluate her goals:

It didn’t come up like “you’re doing this and you should not be doing it” kind of thing, so I think it is negative, it should prompt you to re-evaluate your weight and your goals but it doesn’t, it automatically recalculates your weight, but you can keep changing your end goal and then I’m not sure it brings up like well this is in an underweight category of [Body Mass Index] BMI.

Our health experts also envisioned apps taking more responsibility to protect users, particularly those at risk, by not just focusing on sending notifications to encourage achieving the daily goal but also by notifying when overuse happens:

I think apps could be more intelligent and could give feedback about over-use [...] if you spend too much time on your fitness app or on your diet app or too much time in the gym if you are monitoring all your activities, one could have a warning come up that this might not be the most healthy thing to do.

E-1

On the contrary, the need for a *gentle* approach to encourage engagement was also seen as beneficial:

If someone’s not doing as much physical activity, having an app which kind of brings you back in gently rather than saying “you haven’t exercised for five days, what are you doing,” actually that could be really helpful, so trying to think about encouraging people to do those healthy activities rather than worrying too much about what those outcomes are going to be.

E-3

#### A Need to Reconnect to the Body

Each of the experts highlighted the importance of being able to *listen to your body* and gain the power to *switch off* when needed. They discussed the notion of feeling controlled, or being driven, by apps and how one should have a period of detox if this happens. E-1 highlighted the need for a psycho-educational component about the harms and benefits of healthy eating and fitness apps, explaining that young people in recovery learn to disconnect themselves from their phones:

...trying to live with the anxiety of not knowing the detail of their activity levels and input and outputs; that is something pretty much promoted in recovery.

He further explained how they aim to teach young people to trust their own bodies and themselves, to look after themselves:

It’s natural for one to allow your body to find its own equilibrium and its own input and output in terms of food and activity levels, and to rest when you feel the need to rest and to exercise when you feel the need to exercise.

E-1

This view was also shared by E-2 who explained the need to be mindful around logging behaviors:

Am I checking this because of anxiety and habit or because I want to use it? How autonomous am I in doing this? [...] whether you feel compelled to use it, I think people need to work out whether that’s the case for them or not, like having that awareness is really important and that’s what gives people the power.

E-2

This was echoed by E-3:

I try to think about what am I doing, why am I doing this, what’s this app encouraging me to do, is it actually doing what I want it to be making me do or is it making me do something negative.

E-3

It was acknowledged by E-2 that this can be counter-intuitive to the way healthy eating and fitness apps are currently designed, with users often being actively encouraged to log and monitor themselves constantly: “if you don’t [log] then you mess up your statistics”. He explained how, on one occasion, forgetting his phone at home and not having the ability to track his steps made him feel anxious:

I felt more anxious because I didn’t know how much I’d done and I needed the steps, like the app to tell me that I’d done enough exercise, rather than listening to my body, which I think is a big thing with these apps and social media—they’re prescribed—I think that sort of disconnects people from their bodies a little bit.

E-2

In summary, survey results showed that almost half of our respondents had some form of negative experience using healthy eating and fitness apps. Approximately one-third of respondents reported that they had stopped engaging with healthy eating and fitness apps, typically citing that they found them too demanding or that they lacked the motivation for long-term use. Workshops and interviews further explored personal and professional perspectives on the impact of healthy eating and fitness apps on young people. Participants described how the pressure of attaining health goals, set through the app, could lead to negative experiences and behaviors. There was much consensus that healthy eating and fitness apps can bring feelings of guilt leading to negative experiences and behaviors such as meal skipping. The quantification element of the healthy eating and fitness apps was also seen as a contributing factor for development of maladaptive eating and exercise behaviors, particularly for those with obsessive personalities. Participants concerningly discussed the possible impact of obsession with logging behaviors on social isolation. This was backed up by experts highlighting the importance of being mindful around logging behaviors.

The next stage of our research then focused on exploring the specific features of healthy eating and fitness apps that are currently available on the market, including the ability to log behaviors of interest and how the app responds to user engagement and behavior logging. We wanted to understand the potential of the top 100 healthy eating and fitness apps to elicit the negative experiences and behaviors reported in the first stage.

**Table 4:** Coding criteria and frequency among apps.

Coding criteria		n
**General purpose**		
	Exercise promotion	62
	Diet	10
	Exercise and diet	25
	Appearance goals	65
**Data logged**		
	Exercise completed	84
	Food consumed	23
	Weight	56
**Specific behaviors**		
	Allows underweight goal setting	21
	Allows reporting of high or low calorie intake	16
	Response to low calorie intake	8
	Response to high calorie intake	8
	Response to not meeting exercise goals	20
	Rewards continuous use	60
**Feedback**		
	Overall positive	72
	Overall negative	19
	Achievement-focused	49
	Failure-focused	26
**Data sharing**		
	Other users	32
	Social media	59
	Automatic ranking	6

### Data Analysis: Phase 2

Data from the reviews were collated into a spreadsheet. For review responses that resulted in qualitative data, we performed a qualitative content analysis [62]. As follows, responses were reviewed by 2 members of the research team to identify key themes. Then, the frequency of occurrence of these themes among the apps was coded by these 2 members of the research team individually and summed to produce a score signifying the frequency of that theme with the data set. If there was insufficient information in the text provided in the initial review data to determine whether themes were present in apps, then the coders downloaded the app to clarify whether the app met the coding requirements. Interrater reliability between the 2 coders was again assessed by dividing the number of agreements by the number of disagreements, resulting in an acceptable concordance rate of 90% [36]. The coding criteria developed through this process and frequency of occurrence within apps are shown in Table 4.

For reviews that resulted in quantitative data, responses were summed to produce scores that reflect the number of apps belonging to each possible category. Interrater reliability of this coding had already been determined in the previous review phase (see Table 4).

### Phase 2 Findings: App Review Findings

#### General App Features 

Descriptions of apps were coded according to the health behavior targeted by the app. The vast majority of the apps focused on exercise promotion (n=62) and exercise and diet combined (n=24). A total of 3 apps were found to not focus on either diet or exercise. This included 1 app that focused on editing the body to assess how one would look like with various bodily enhancements (Body Editor–Breast Enlarger, Body Shape Editor) and 2 that focused on weight monitoring (eg, Monitor your Weight). Around two-thirds of the apps included the ability to set appearance-related goals, such as weight loss and enhancement in muscle tone (n=65).

The majority of apps collected some form of data pertaining to physical activity (n=84). This included data such as daily steps, cycling, and the completion of workouts specified by the app. A much smaller percentage of apps collected dietary data pertaining to food consumption (n=23). More than half requested user data pertaining to weight (n=56).

#### Specific Behaviors

In total, 21 apps allowed the setting of underweight body goals (corresponding to a BMI<18.5). Qualitative responses to other questions highlighted how this could include setting extremely low BMI targets in some apps: “I still can set a BMI goal<13”.

Around two-thirds of the apps that facilitated the recording of dietary intake allowed the reporting of calorie consumption that was 50% more or 50% less than the recommended daily amount for the average individual (n=16). Some apps responded differently to the entering of low-calorie consumption (n=8), for example, highlighting that a goal had not been met or not allowing the calories to be logged. Others had a minimum calorie entry threshold and would not allow calories to be logged under this target. Some apps treated the under logging of calories the same as logging calorie content in line with their goals (n=8), including those that praised under logging calories in the same way that they praised calorie entry consistent with goals (eg, “You made that look easy”—Lose it!). A small proportion of apps also responded differently to logging calories that exceeded the daily target (n=8). Example responses to exceeding calorie goals included numeric feedback, reminders about goals, and visual feedback such as turning the calorie counter red or sad face emoji. Furthermore, some apps had a more questioning approach, for example, asking if the calorie entry is correct.

A fifth of the apps responded to failure to meet exercise goals (n=20). The nature of these responses varied and included reminders, numerical feedback. Similar to when logging a high calorie intake, some apps adopted a questioning approach (“Wanna give up? Think about why you started?”). Two-thirds of the apps offered praise and rewards for continuous use (n=60). Qualitative responses as to the nature of this praise varied. Many offered direct messages of praise such as congratulations messages or fireworks. Elements of gamification were present in some apps, with trophies, badges, and the unlocking of levels being used to reward continuous use.

#### Feedback

Apps were coded according to whether positive or negative feedback was used to motivate users’ engagement with the app. The majority focused on positive feedback (n=72), whereas a minority of apps were felt to focus on negative feedback (n=19). The remainder of apps either provided neutral feedback (n=8) or provided both positive and negative feedback to the extent that the reviewers could not differentiate between which was being used more in the app (n=1).

Examples of both positive and negative feedback were provided in qualitative responses to other questions (eg, how the app responds to specific user behaviors). Some apps sent positive feedback to encourage users to keep up with their initial goal “Hey [user name]! You’ll feel great after a workout. Strive for progress, not perfection” or suggested that the user adjust their personal goal so that it was more in line with their lifestyle “You haven't been active lately, do you want to adjust your goal”. An example of negative feedback included users being punished for not reaching their personal target “if you miss a day you'll get punished by losing a heart”.

Furthermore, apps were coded as to whether they focus on what the user has achieved or what they have not achieved. Just under half of the apps were positively focused on achievements (n=49), highlighting what the user had done, whereas around one quarter were negatively focused on achievements (n=26), highlighting what the user had not done. The remainder did not focus on achievements (n=25) and included apps that did not involve goal setting or targets and those with predetermined reminders not linked to user behavior.

#### Data Sharing

Around one-third of apps facilitated the sharing of data with other app users (n=32). The remaining apps either did not facilitate this (n=66) or did not collect any user data (n=2). A much higher proportion of apps facilitated the sharing of app-related achievements through social media sites (n=59), whereas the remainder did not (n=39) or did not collect any data (n=2).

A small proportion of apps automatically ranked users among others (n=6) but all of these apps allowed this feature to be deactivated. This ranking approach was evident in the qualitative responses about app feedback. For example, a push-up workout app compared user reports about their workouts with other users in similar categories (based on BMI, gender, and age): “you did X push-ups, this is better than XX% of users today”.

In summary, we wanted to identify the logging functionalities available in the top 100 healthy eating and fitness apps that have the potential to elicit negative experiences and behaviors captured in the first stage. We found that apps responded differently to data-logging behaviors, for example, some allowing users to under log their calorie consumption whereas others using guilt-inducing techniques when calories exceed the daily goal. Alarmingly, 21% of the apps we reviewed allowed underweight goal setting (BMI<18.5). In addition, one quarter of the apps focused on negative reinforcement for maintaining health goals. A substantial number of apps facilitate sharing data with other users (n=32) and across social media (n=59).

## Discussion

### Principal Findings

The aim of this research was to understand the potential role of healthy eating and fitness apps in the development of maladaptive body-related attitudes and eating and exercise behaviors in young people. Drawing on the findings from Phase 1 and Phase 2, we have identified 5 important ways in which healthy eating and fitness apps may potentially exert negative impacts on users. In this section, we reflect on these themes and how future research may mitigate against these issues.

### The Attraction of the Number Game

Calorie counting is a prominent feature of many currently available healthy eating and fitness apps and a key reason why many of the survey respondents, both male and female, reported using healthy eating and fitness apps. However, participants in both the survey and workshops also described how obsessive thoughts and behaviors around calorie counting and logging could emerge. For example, not achieving a calorie count within a certain boundary could cause feelings of failure and guilt, often then leading to restrictive eating or excessive exercising behaviors (eg, not allowing themselves to eat after 3 pm), simply because the app indicated that they had already reached their caloric intake for the day. This tendency to engage in purge behaviors echoes findings from previous research examining the consequences of healthy eating and fitness app use [32] and food journaling [20,63].

In addition to calorie counting, there were other aspects of self-tracking evident in the apps reviewed (eg, exercise tracking and weight tracking). The primary objective of self-tracking is around obtaining *self-knowledge through numbers* [64], and these types of self-tracking practices are used successfully by large numbers of people to achieve their goals. Indeed, participants in both our study and previous research have expressed experiencing acute benefits from short-term self-tracking [28]. However, the act of self-tracking was often seen as a *numbers game* for the young people in our study, becoming a law for them to live their lives by. In the workshops, participants describe how self-tracking led them to over focus on the calories contained in food at the expense of nutrition (eg, prioritizing ready meals as this was easier to log). Furthermore, some survey respondents described cheating the app by not accurately logging their food consumption. Although healthy eating and fitness apps offer new ways of monitoring, measuring, and representing the human body [64], they also contribute to a new way of conceptualizing one’s health status and introduce a new language around digitizing one’s health, making it easier to trust *the numbers* over physical observations [64]. This obsession with numbers can facilitate a shift toward unhealthy behavior in contrary to the healthy eating and fitness apps’ initial goal of promoting health.

Reflecting on these points, we need to think about better ways to log food and exercise. For example, [65] have used picture logging of food plate for older people to help with monitoring of nutritional value of daily food intake in the care home environment. In the context of self-tracking, [66] also have investigated photo-based journals as a simplified self-tracking method. This style of easy logging and calorie estimation might be a more forgiving approach to take.

Furthermore, our experts highlighted a need for approaches that promote listening to one’s own body, and its nutritional needs and physical limitations, rather than becoming reliant on the attainment of what are often arbitrary numerical goals. Such an approach is consistent with newly emerged evidence-based psychological approaches to improving exercise and eating behavior that are focused on the development of embodied and mindful approaches to eating and exercise [67]. Reframing the way that young people think about eating and exercise, by moving away from language embedded in self-quantification and self-objectification, could be an important first step toward understanding how we might achieve this within future technologies that support healthy eating and fitness.

### Over Emphasis on Extrinsic Appearance (Including Weight Loss) Goals

A high percentage of healthy eating and fitness apps (almost two-thirds) currently available on the market focus on the achievement of appearance-related goals rather than health goals. This finding is consistent with research conducted around other health and fitness media, such as magazines [68].

Focusing on appearance-related goals in eating and exercise settings has been linked to negative body image and maladaptive eating and exercise behavior [69]. In contrast, appreciating the functionality of one’s body rather than its extrinsic appearance can enhance well-being and reduce vulnerability to environmental triggers of body dissatisfaction [70,71]. Similarly, focusing on more intrinsic factors when eating and exercising is more conducive to long-term physical and mental health [72,73]. Future apps that focus more on the intrinsic value of exercise and eating behaviors (eg, how food and exercise makes us feel, how much enjoyment we get from the experience) rather than extrinsic weight loss and appearance could be a powerful tool to supporting healthier attitudes toward food and exercise.

Relatedly, and of particular concern, is the fact that around a fifth of the apps we reviewed allowed underweight goal setting. As this is a relatively underresearched area, it is unclear the extent to which the ways that an app’s reaction to underlogging of calories might affect individuals (eg, if a user is being notified that they are not reaching their underweight goals or if they are trusting that a healthy eating and fitness app would not allow than to set an underweight goal in the first place). However, it is possible that these types of responses might serve as *thinspiration* (ie, digital content that is ostensibly designed to inspire thinness and is commonly found in Web-based eating disorder communities [74]), motivating weight loss among individuals who already display problematic attitudes toward eating and exercise. Future research should examine this further; however, a simple safety measure could be implemented to ensure that users are unable to set unrealistic underweight goals (ie, those that would result in a BMI less than 18.5) in the first place and that they are provided with relevant information about the dangers of setting underweight goals if they attempt to do so.

### The Power of Notifications

The literature on behavior change, and the roles of notifications in supporting change, is extensive [35,75,76]. Indeed, in our study, we found that participants appreciated notifications, particularly those containing positive content, and generally considered them to be a motivational tool. However, our app review also, concerningly, saw several examples of apps that were seen to enact punishments on the users or act as a constant reminder of the failure to achieve daily goals. In the context of body-modification strategies for weight loss or appearance-enhancement motives, guilt is generally regarded as problematic [77].

In our study, young people openly discussed the negative emotions they have experienced when interacting with healthy eating and fitness apps. They expressed feelings of guilt, disappointment, and pressure. This echoes findings from [28,63], where feelings of guilt were associated with not achieving goals.

Although the aim of notifications is to encourage engagement, previous research suggests that these are not always effective and can instead cause additional stress on young people [63]. Rather than focusing on temporal reminders to complete a daily task, apps should encourage a process of self-reflection and habit formation. For example, users could be encouraged to think in advance about occasions that could interrupt the attainment of daily goals, facilitating a personalized approach for allowing users to plan strategies for how to cope when this happens. Following a preplanned strategy would avoid *extreme measure* behaviors (eg, not eating anything for the rest of the day once calorie allowance has been reached), instead promoting better coping mechanisms (eg, if a workout is missed in the evening because a user is trying to meet a deadline, they can plan to conduct the exercise the next day). Consequently, engaging users in the creation of preplanned statements that mitigate negative feelings such as guilt triggered by not meeting goals might be a useful way of supporting users.

### Social Impact

Healthy eating and fitness apps offer the opportunity to enhance the social life of the user by giving access to group memberships and opportunities to find like-minded people or friends on the internet (sharing and comparing data through the app) and offline (engaging in physical activities with groups of preexisting friends or new ones through the app) [78]. Many apps provide the opportunity for users to rank themselves among other users, thus encouraging competition. For example, FitBit has a community section where users can add each other as friends and *compete* toward weekly step counts. Research suggests that perceived social norms can be a powerful motivator of behavior change in relation to eating and exercise, in that individuals are more likely to engage in behavior if they perceive others to be doing it [42]. However, comparing the self with others can also be demotivating and detrimental to self-perceptions, especially if users perceive themselves as comparing unfavorably with others [33].

Our app review indicates that the majority of apps facilitated social features, that is, data sharing with other app users (n=32) or through social media (n=59). However, although healthy eating and fitness apps are seen as socially mediated experiences to connect people with common interests and goals for healthy eating and physical activity [78], our qualitative data suggested that individual app engagement can result in antisocial behavior (eg, not attending social functions) that can lead into loneliness and impact the psycho-social development of a young person, for whom the development of social relationships is an important and psychologically salient developmental task [79]. Reflecting on these points, it is important that future healthy eating and fitness apps not only facilitate creation of a digital connection and communities but also provide tools to encourage social connectivity. This might be through the provision of a space to reflect on their social interactions or by providing them with practical tips and strategies (eg, advance planning for social occasions or integrating healthy eating and fitness activities in people’s social lives).

### Consequences of Sustained Use

Most healthy eating and fitness apps are designed to reinforce continuous use. Typically, this is achieved through gamification features that render daily routines into games [80]. Gamification is defined as the use of game design elements (eg, game-like rewards, leaderboards [81], and storytelling [82]) in nongame context (eg, healthy eating behavior) [83]. Gamification is increasingly being used in healthy eating and fitness apps, yet the effectiveness of this technique has only been assessed against revenue generation and not long-term behavior change [84].

Although the aim of gamifications is to promote sustained use, studies have shown that long-term engagement is not always achievable, for example, only 11.6% of app users in Great Britain are still engaging with an app a week after installing it on their phones (findings from other countries are similar) [85]. Our survey findings indicate that most app users do not report long-term engagement with apps, typically withdrawing from apps because of finding them too demanding, lacking motivation, boredom, annoyance of notifications, or feeling negative emotions when failing to meet the set targets. This was further supported in the workshops where participants described how the reinforcement of continuous use can have unintended negative consequences, such as experiencing guilt and experiencing the desire to stop using the app entirely.

One possible option for the future app developer would be to consider encouraging more long-term behavior change, rather than focusing on engagement [86]. Furthermore, given that obsessive engagement with apps may reflect problematic use, apps could provide informative feedback based on usage patterns (eg, frequency of app checking or time spent engaging with app) that might be indicative of problem use. For example, to overcome both the obsession and indeed lack of motivation and boredom, the apps could encourage a *period of detox* when unusual patterns of use are detected, as part of the gamification model. In addition, providing a framework within the app that encourages users to self-reflect on their use patterns and to re-evaluate their goals regularly may help users who might be struggling to re-gain a sense of much needed control.

### Limitations

This research adopted a mixed-methods approach to develop a holistic understanding of the potential negative impact of healthy eating and fitness app use among young people. Though we aimed to recruit both male and female participants to engage in our qualitative research, we struggled to recruit individuals identifying as male to engage in workshop activities. That said, male perspectives are still represented in the survey data and in fact made up the majority of respondents. One reason for this could be that we recruited largely from computer science, which is a predominantly male field; however, for future work, a gender balance would be preferable. We also did recruit a male eating disorder campaigner, activist, and writer with a personal experience of eating disorder who advocates for greater recognition of eating disorder service delivery and the male experience to participate in the interviews. However, future research should aim to more deeply explore the male experience of healthy eating and fitness app use further. Similarly, it may be important for future research in this field to consider how other individual difference variables, such as race, class, body size, and disability, affect participants’ engagement with healthy eating and fitness apps and their potential for misuse.

### Conclusions

Through our study, we have offered a deepened understanding of young people’s experiences of healthy eating and fitness apps and the potential harm that their use might have. We have offered a set of guidelines for future apps that can be responsibly developed to prevent the formation of maladaptive eating and exercise behaviors. Although we understand that an app developer would never set out to meaningfully bring about negative emotional responses in their users, we must also be mindful of the fact that these responses are happening. Through this study, we hope to open a dialogue around how use of these apps could have the potential to become the seed that develops into a more serious issue. As the target user demographic for these types of apps, young people are most vulnerable to the development of poor body image and maladaptive eating and exercise patterns. As such, we need to take care in the type of language we are using and the type of sustained behaviors we are promoting.

## Abbreviations

BMI: body mass index
